# Recurrent post-partum coronary artery dissection

**DOI:** 10.1186/1749-8090-5-78

**Published:** 2010-10-09

**Authors:** Taufiek K Rajab, Zain Khalpey, Bernhard Kraemer, Frederic S Resnic, Robert P Gallegos

**Affiliations:** 1 Division of Cardiac Surgery, Department of Surgery, Brigham and Women's Hospital, Harvard Medical School, Boston, MA 02115, USA; 2 Department of Obstetrics and Gynecology, University of Tuebingen, 72076 Tuebingen, Germany; 3 Department of Cardiovascular Medicine, Brigham and Women's Hospital, Harvard, Medical School, Boston, MA 02115, USA

## Abstract

Coronary artery dissection is a rare but well-described cause for myocardial infarction during the post-partum period. Dissection of multiple coronary arteries is even less frequent. Here we present a case of recurrent post-partum coronary artery dissections. This unusual presentation poses unique problems for management. A 35 year-old female, gravida 3 para 2, presented with myocardial infarction 9 weeks and 3 days post-partum. Cardiac catheterization demonstrated left anterior descending (LAD) dissection but an otherwise normal coronary anatomy. The lesion was treated with four everolimus eluting stents. Initially the patient made an unremarkable recovery until ventricular fibrillation arrest occurred on the following day. Unsynchronized cardioversion restored a normal sinus rhythm and repeat catheterization revealed new right coronary artery (RCA) dissection. A wire was passed distally, but it was unclear whether this was through the true or false lumen and no stents could be placed. However, improvement of distal RCA perfusion was noted on angiogram. Despite failure of interventional therapy the patient was therefore treated conservatively. Early operation after myocardial infarction has a significantly elevated risk of mortality and the initial dissection had occurred within 24 hours. This strategy proved successful as follow-up transthoracic echocardiography after four months demonstrated a preserved left ventricular ejection fraction of 55-60% without regional wall motion abnormalities. The patient remained asymptomatic from a cardiac point of view.

## Background

Myocardial infarctions in women of childbearing age are rare. Myocardial infarctions related to pregnancy are even less common, occurring with an incidence of approximately 6 per 100,000 as estimated by a US population-based study [1]. During the post-partum period, coronary artery dissection is the prime cause for myocardial infarction [2,3]. The first case report of idiopathic coronary artery dissection was described in 1931 [4]. Subsequently 83 cases of pregnancy-associated coronary artery dissection were reported in a review of the literature until the year 2009 [5]. Since then we have identified an additional 5 case reports of pregnancy-associated coronary artery dissection [6-10]. However, dissection of multiple coronary arteries occurred only in a very small subset of the previously published cases [5]. Here we present a patient with recurrent dissection of multiple coronary arteries. This unusual presentation poses unique problems for management.

## Case presentation

A 35 year-old female, gravida 3 para 2, presented to the emergency department with her first ever episode of angina pain 9 weeks and 3 days following an uneventful caesarian section. The patient noted constant chest tightness with radiation to both arms while getting ready for work. The pain was associated with diaphoresis but she denied dyspnea or nausea. Six years prior she underwent catheter pulmonary embolectomy for a thromboembolism thought to be related to oral contraceptive use. Since then she had been taking warfarin and warfarin was restarted postpartum. Otherwise the past medical history was only significant for hypertension. The family history was notable for a younger sister who was diagnosed with cardiomyopathy six weeks postpartum and a grandmother who died of unknown causes suddenly at age 42 without a relationship to pregnancy.

**Figure 1:**
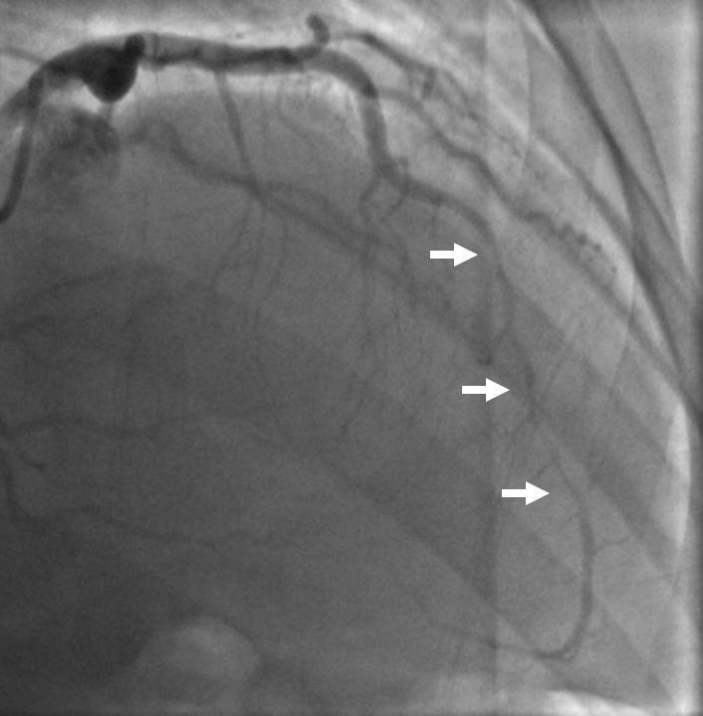
**Angiographic view showing LAD dissection 67 days post-partum involving the mid vessel (arrows)**. There is TIMI-2 flow distally.

**Figure 2:**
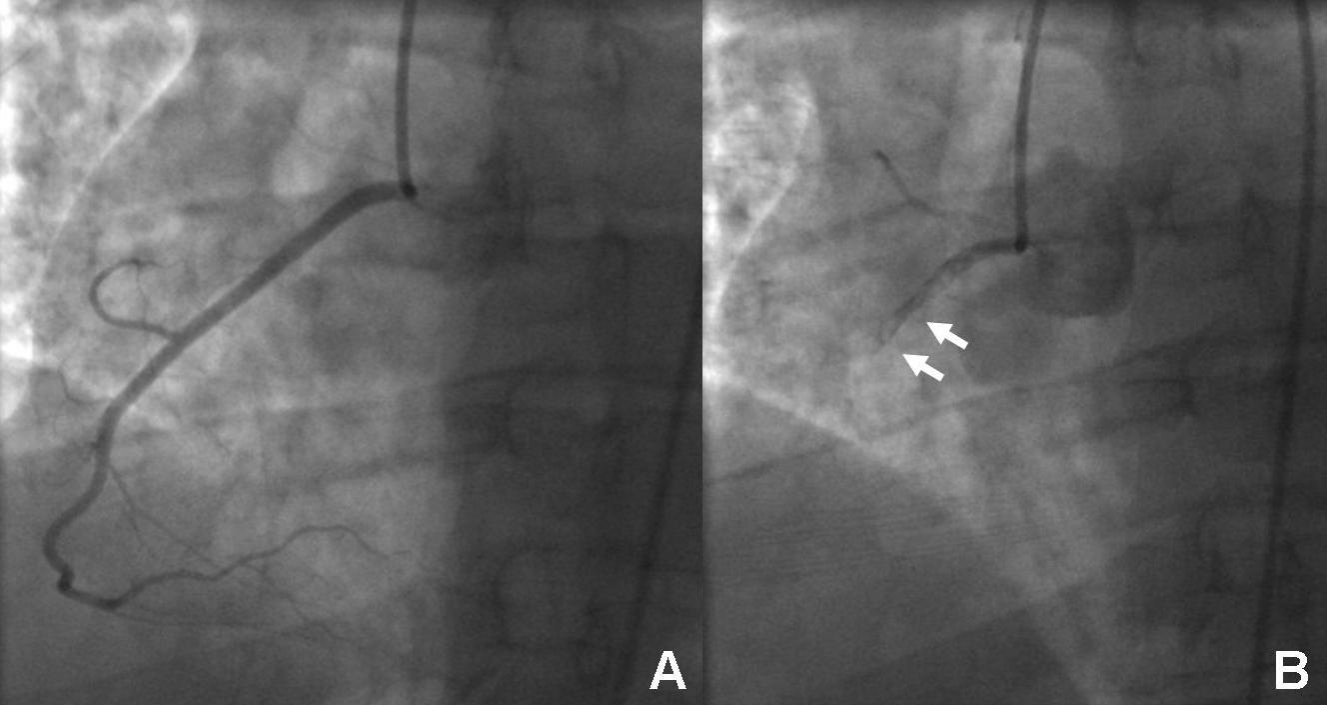
Panel A shows the normal RCA 67 days post-partum, Panel B shows recurrent dissection on repeat angiography 68 days post-partum.

**Figure 3:**
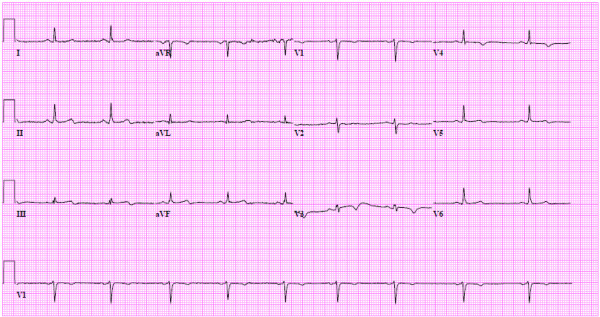
**Post-intervention EKG demonstrates no evidence of RCA territory ischemia**.

**Figure 4:**
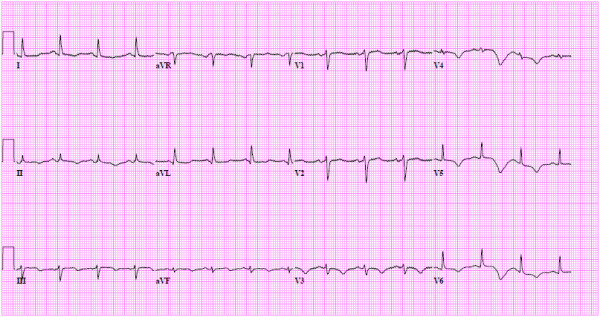
**The EKG demonstrates new T wave inversion in the lateral leads**.

Upon physical examination the pulse rate was 83, respiratory rate 16 and blood pressure 130/67. The EKG demonstrated evidence of anterior ischemia. Serial troponin-t peaked at 1.17 ng/mL. The INR measured 1.9 IU. Initial treatment consisted of loading with 300mg clopidogrel. Emergent cardiac catheterization showed left anterior descending (LAD) coronary artery dissection complicated by extensive thrombus (Figure 1, Additional file 1). Otherwise the coronary anatomy was without lesions in the left main coronary artery, the circumflex coronary artery or the right coronary artery (RCA) (Figure 2 Panel A, Additional file 2). The dissection affected the mid LAD to the distal LAD with irregular severity. Intravascular ultrasound demonstrated subintimal thrombosis but there was no evidence of a free dissection plane. Three everolimus eluting stents (Xience, Abbott Laboratories, USA) measuring 2.5 × 18 mm, 2.5 × 23 mm and 2.5 × 18 mm were deployed and dilated to 3.5 mm proximally. However, an edge dissection of the most distal stent became apparent after treatment of the target lesion. This was covered with an additional 2.5 × 18 mm stent. Notably, the post-intervention EKG demonstrated no evidence of ischemia in the RCA territory (Figure 3). The post-intervention course was unremarkable until a witnessed episode of ventricular fibrillation arrest occurred the following day. Cardiopulmonary resuscitation was undertaken for 7 minutes and unsynchronized cardioversion with 200 joules restored a normal sinus rhythm. The EKG showed new T wave inversion in the lateral leads (Figure 4). Upon repeat catheterization it was discovered that her non-dominant RCA had newly dissected and was occluded with thrombus (Additional file 3). A wire was passed distally, but it was unclear whether this was through the true or false lumen and no stents were placed. However, improvement of distal perfusion was noted on angiogram (Figure 2 Panel B, Additional file 4). In view of this, as well as the recent myocardial infarction, the patient was treated conservatively. Transthoracic echocardiography the following day demonstrated a low normal left ventricular ejection fraction of 50-55% as well as apical hypokinesis. This was confirmed by cardiac magnetic resonance imaging. She was discharged five days later on aspirin, warfarin, prasugrel, metoprolol, atorvastatin, and magnesium oxide. The patient made an un-eventful further recovery. Follow-up transthoracic echocardiography after four months demonstrated an improved left ventricular ejection fraction of 55-60% without definite regional wall motion abnormalities. The patient remains asymptomatic.

## Discussion

We present a case of recurrent coronary artery dissections, which were treated conservatively. The case is notable in three respects. Firstly, recurrent post-partum coronary artery dissection is extremely unusual. Secondly, the patient presented 9 weeks and 3 days post-partum, which is relatively late compared to previously described cases [11]. Thirdly, the recurrence was complicated by failure to place a coronary artery stent, which presented unique problems for management of this dissection. The specific predisposing factors of the peripartum period in the pathogenesis of spontaneous coronary artery dissection are still unclear. Most coronary artery dissections occur within two weeks post-partum [11]. This indicates that physiological factors related to parturition are associated with a propensity for coronary artery dissection. Our patient presented over two months post-partum, which is relatively late. Shah and colleagues described a 23-year-old patient who also presented with coronary artery dissection that occurred two months after elective abortion at 14 weeks [12]. This patient would later also develop recurrent dissection. The reason why patients are susceptible to recurrent dissection of multiple coronary arteries such a long time after parturition is not clear and could be a genetic predisposition.

Possible treatment strategies for coronary artery dissections are medical therapy, coronary intervention and coronary artery bypass surgery (CABG) [5,13]. There are no randomized trials comparing these treatment options and the optimal therapeutic strategy is not clearly defined. Medical therapy alone is an option for hemodynamically stable patients with adequate coronary blood flow and no signs of persistent ischemia. Coronary artery intervention is indicated for patients with ongoing signs of ischemia. Finally, the indications for coronary artery bypass grafting include involvement of the left main coronary artery, multi-vessel dissection and failure of interventional therapy. In the described case, initial dissection of the LAD was treated with everolimus eluting stents. Drug-eluting stents provide inhibition of neointimal proliferation, which occurs as a result of vascular injury. Therefore drug eluting stents were chosen over bare metal stents. When recurrent myocardial infarction was diagnosed the patient was emergently taken to the catheterization lab. This demonstrated new dissection of the previously normal RCA and a wire was passed distally but it was unclear whether this was through a true or false lumen. Therefore no stent could be placed. Therefore surgical therapy to treat the RCA lesion was considered. However, the patient had undergone myocardial infarction on the previous day due to LAD dissection. Notably, early operation after myocardial infarction carries a significantly elevated risk of mortality [14,15]. Furthermore distal RCA blood flow was evident (Figure 2 Panel B). Therefore the second dissection was treated conservatively rather than by CABG. The conservative management strategy was effective as the patient has remained asymptomatic to followup. Echocardiography four months after the myocardial infarctions showed improved left ventricular function with an ejection fraction of 55-60%. No repeat coronary catheterization was undertaken because the patient was asymptomatic. In our opinion, repeat dissection of the RCA represented spontaneous recurrent postpartum coronary dissection. Coronary artery dissection can also be a complication of angiography. Iatrogenic coronary artery dissection occurs in 0.03-0.06% of diagnostic catheterizations [16]. Risk factors include catheterization for acute myocardial infarction, atherosclerosis, hypertension and vigorous contrast injection [17]. Acute myocardial infarction and hypertension were present in the patient. However, iatrogenic catheterinduced coronary dissection occurs at the time of catheterization. The post-intervention EKG in our patient demonstrated no evidence of ischemia in the RCA territory (Figure 3). Thus, iatrogenic catheter-induced RCA dissection can be ruled. In contrast, she developed recurrent myocardial infarction with new changes in the RCA territory one day after the original LAD dissection. This is explained by de-novo dissection of the RCA.

## Conclusion

We present a case of recurrent post-partum coronary artery dissections. This presentation is highly unusual, and no guidelines exist whether management should be conservative or surgical. While there are some indications for CABG surgery we decided to pursue a conservative strategy with coronary artery stenting of the first dissection and medical management of the second dissection despite the inability to stent the second lesion. This strategy proved successful.

## Consent

Written informed consent was obtained from the patient for publication of this case report and accompanying images. A copy of the written consent is available for review by the Editor-in-Chief of this journal.

## Competing interests

The authors declare that they have no competing interests.

## Authors' contributions

TKR, ZK, RSF and RPG were involved in the patient's clinical care. TKR wrote the manuscript, which was critically revised for important intellectual content by ZK, BK and RPG. All authors read and approved the final manuscript.

## Supplementary Material

Additional file 1 **Supplementary video showing initial left heart catheterization with dissected LAD**. Click here for file

Additional file 2 **Supplementary video showing initial right heart catheterization with normal RCA**. Click here for file

Additional file 3 **Supplementary video showing repeat right heart catheterization with dissected RCA**. Click here for file

Additional file 4 **Supplementary video showing repeat right heart catheterization after revascularization**. Click here for file

## References

[B1] James A Jamison M Biswas M Brancazio L Swamy G Myers E Acute myocardial infarction in pregnancy: a United States population-based study Circulation 2006 113 12 1564 71 10.1161/CIRCULATIONAHA.105.576751 16534011

[B2] Roth A Elkayam U Acute myocardial infarction associated with pregnancy Ann Intern Med 1996 125 9 751 62 8929010 10.7326/0003-4819-125-9-199611010-00009

[B3] Roth A Elkayam U Acute myocardial infarction associated with pregnancy J Am Coll Cardiol 2008 52 3 171 80 10.1016/j.jacc.2008.03.049 18617065

[B4] Petty HC Dissecting aneurysm of coronary artery in a woman aged 42: rupture Br Med J 1931 1 667 10.1136/bmj.1.3667.667

[B5] Appleby C Barolet A Ing D Ross J Schwartz L Seidelin P Contemporary management of pregnancy-related coronary artery dissection: A single-centre experience and literature review Exp Clin Cardiol 2009 14 1 e8 e16 19492033 PMC2689090

[B6] Al-Mohaissen M Spontaneous left main coronary artery dissection. A rare cause of acute coronary syndrome Saudi Med J 2009 30 11 1476 9 19882065

[B7] Karadag B Roffi M Postpartal dissection of all coronary arteries in an in vitrofertilized postmenopausal woman Tex Heart Inst J 2009 36 2 168 70 19436817 PMC2676589

[B8] Topal A Eren M Acute ventricular rupture due to myocardial infarction during postpartum period Interact Cardiovasc Thorac Surg 2009 8 5 565 7 10.1510/icvts.2008.189175 19237401

[B9] Rahman S Abdul-Waheed M Helmy T Huffman L Koshal V Guitron J Spontaneous left main coronary artery dissection complicated by pseudoaneurysm formation in pregnancy: role of CT coronary angiography J Cardiothorac Surg 2009 4 15 19338659 10.1186/1749-8090-4-15 PMC2669072

[B10] Collyer M Bellenger N Nachimuthu P Parasuraman R Taylor M Postpartum coronary artery dissection J Obstet Gynaecol 2008 28 4 451 3 10.1080/01443610802163633 18604695

[B11] Koul A Hollander G Moskovits N Frankel R Herrera L Shani J Coronary artery dissection during pregnancy and the postpartum period: two case reports and review of literature Catheter Cardiovasc Interv 2001 52 1 88 94 10.1002/1522-726X(200101)52:1<88::AID-CCD1022>3.0.CO;2-P 11146532

[B12] Shah P Dzavik V Cusimano R Sermer M Okun N Ross J Spontaneous dissection of the left main coronary artery Can J Cardiol 2004 20 8 815 8 15229764

[B13] Creswell L Moulton M Cox J Rosenbloom M Revascularization after acute myocardial infarction Ann Thorac Surg 1995 60 1 19 26 7598589

[B14] Voisine P Mathieu P Doyle D Perron J Baillot R Raymond G Influence of time elapsed between myocardial infarction and coronary artery bypass grafting surgery on operative mortality Eur J Cardiothorac Surg 2006 29 3 319 23 10.1016/j.ejcts.2005.12.021 16439152

[B15] Weiss E Chang D Joyce D Nwakanma L Yuh D Optimal timing of coronary artery bypass after acute myocardial infarction: a review of California discharge data J Thorac Cardiovasc Surg 2008 135 3 503 11 11.e1-3 10.1016/j.jtcvs.2007.10.042 18329460

[B16] de Bono D Complications of diagnostic cardiac catheterisation: results from 34,041 patients in the United Kingdom confidential enquiry into cardiac catheter complications. The Joint Audit Committee of the British Cardiac Society and Royal College of Physicians of London Br Heart J 1993 70 3 297 300 10.1136/hrt.70.3.297 8398509 PMC1025320

[B17] Boyle A Chan M Dib J Resar J Catheter-induced coronary artery dissection: risk factors, prevention and management J Invasive Cardiol 2006 18 10 500 3 17015916

